# Ultrasound-Guided Percutaneous Neuromodulation in Patients with Chronic Lateral Epicondylalgia: A Pilot Randomized Clinical Trial

**DOI:** 10.3390/ijerph18094877

**Published:** 2021-05-03

**Authors:** Blanca De-la-Cruz-Torres, Vanesa Abuín-Porras, Emmanuel Navarro-Flores, César Calvo-Lobo, Carlos Romero-Morales

**Affiliations:** 1 Department of Physiotherapy, University of Seville, Avicena Street, 41009 Seville, Spain; 2 Faculty of Sport Sciences, Universidad Europea de Madrid, Villaviciosa de Odón, 28670 Madrid, Spain; vanesa.abuin@universidadeuropea.es (V.A.-P.); carlos.romero@universidadeuropea.es (C.R.-M.); 3 Faculty of Nursing and Podiatry, Department of Nursing, University of Valencia, Frailty Research Organizaded Group (FROG), 46010 Valencia, Spain; emmanuel.navarro@uv.es; 4 Facultad de Enfermería, Fisioterapia y Podología, Universidad Complutense de Madrid, 28040 Madrid, Spain; cescalvo@ucm.es

**Keywords:** lateral epicondylalgia, radial nerve, ultrasound, chronic pain, neuromodulation, percutaneous nerve stimulation, rehabilitation

## Abstract

Objective: The aim was to analyze effects of a percutaneous neuromodulation (PNM) treatment on the radial nerve, regarding pain, functionality, electrophysiologic excitability, and morphology, in patients with chronic lateral epicondylalgia (LE). Methods: Twenty-four patients with chronic unilateral elbow pain were recruited for this preliminary study and were divided into two groups: control (n = 12) and PNM group (n = 12). The subjects in the PNM group received percutaneous peripheral neurostimulation with an acupuncture needle that was located next to the nerve with ultrasound guidance. Pain using a numerical rating scale (NRS), functional ability using patient-rated tennis elbow evaluation (PRTEE), radial nerve cross-sectional area measured by ultrasound, and chronaxie and accommodation index (AI) measured by the strength–duration curve were evaluated. Results: Both groups showed no differences in the baseline measurements (all *p* = 0.001). However, at the end of the treatment, there were significant differences between groups since only the PNM group significantly improved their values compared to their baseline values: level of pain and cross-sectional area (CSA) values showed a significant decrease while the PRTEE scores showed a significant improvement. Then, regarding AI, the PNM group showed significant improvement for the electrophysiologic nerve excitability pattern, reporting normal function in all radial nerves after treatment (*p* = 0.001). However, chronaxie values always reported similar values with no differences between groups (*p* >0.05); Conclusion: Ultrasound-PNM technique may be an interesting therapeutic tool for the treatment of chronic LE due to the improvement in the level of pain, functionality, nerve morphology, and excitability in this population.

## 1. Introduction

Lateral epicondylalgia (LE) is characterized by pain at the lateral epicondyle of the humerus aggravated by dorsiflexion and/or supination of the wrist against resistance. LE is described as the most common injury of the elbow, affecting 1–3% of society [1]. The underlying lesional mechanism in LE is a fibroblastic and vascular response but not an inflammatory response [2]. This process is based on an interruption of collagen fibers with few inflammatory cells [3,4]. In refractory LE, Gürcay et al. [5] showed that the radial nerve is swollen on the affected sides and De-la-Cruz-Torres [6] found that patients with chronic LE had decreased excitability in any radial nerve, independently of the affected limb.

Hence, the selection of an effective intervention could help to manage the condition and at the same time lessen the financial burden, which is important. In addition, passive physical modalities, including electrotherapy and orthotic devices, are common treatments for the management of elbow pain [7,8]. Moreover, these approaches involve a device that does not require active participation by the patient. In a systematic review, in 2017, Dion et al. [9] examined the effectiveness of passive physical modalities for the treatment of soft-tissue injuries of the elbow, but little evidence exists to support or refute their use. According to the best of the authors’ knowledge, there is evidence on the medical treatment [10,11,12], but there are limited studies on the physiotherapeutic treatment of the radial nerve in LE [13,14].

Since its first description by Wall and Sweet in 1967 [15], peripheral nerve stimulation (PNS) efficacy for chronic neuropathic pain in the upper limbs is controversial [16,17,18,19]. The analgesic effect induced by low-frequency PNS may be due to the combination of peripheral and central mechanisms. This current preferentially excites large myelinated fibers: modulating peripheral nociceptors, [20] blockading afferent nociceptive inputs by continuous stimulation [21] and modulating nociception in the dorsal horn of the spinal cord [21]. From the medical perspective, many PNS techniques have been applied for the treatment of chronic neuropathic pain located in the upper limb, targeting either peripheral nerve trunks or nerve roots within the brachial plexus [16,17,18,19]. In these techniques, patients underwent permanent placement of a percutaneous lead and an implant of an implantable pulse generator. In this line, physiotherapists are clinically applying a minimally invasive procedure, known as ultrasound (US)-guided percutaneous neuromodulation (US-guided PNM), [22] that consists in the application of percutaneous neurostimulation with an acupuncture needle that is located next to the nerve or motor point of the muscle with US guidance [22]. This is an accessible, invasive, safe and economical procedure to perform for the treatment of neuropathic pain by therapists. To date, there is limited literature on US-guided PNM. It has been applied to treat lateral elbow pain [14], short hamstring syndrome [23], anterior knee pain [24,25], or lower back pain [26]; to improve the activity of the hallucis flexor longus [27,28] or quadriceps strength [29]; and to improve sport performance [30]. Regarding LE, Arias-Buría et al. [14] described a case-report and they only treated a patient with LE by applying PNM to analyze its effect on pain. Researchers hypothesize that there may be structural and excitability changes at the nerve level as a consequence of decreased nerve sensitization and the PNM treatment.

Authors suggested that different neuromodulation procedures have been described in the health sciences, but the PNM technique is a recently developed invasive physiotherapy tool that may be used in a simple and careful way by physiotherapists in therapeutic centers. The objective of this preliminary study was to analyze the effects of PNM treatment on the radial nerve, regarding pain, functionality, electrophysiologic excitability, and morphology, in patients with unilateral chronic LE. Findings from this study may provide further evidence of the relevance of neural tissues in determining elbow pain and may indicate the effects of the US-guided PNM technique on rehabilitation and prevention in patients with unilateral LE.

## 2. Methods

### 2.1. Design

The study was a pilot randomized clinical trial and was carried out from 1 March to 20 June 2018. It was performed to adjust the sample size for a larger clinical trial. Hence, 24 patients were recruited. The entire recruiting process was carried out in a private sport center.

### 2.2. Participants

Twenty-four subjects with unilateral pain in the lateral aspect of the elbow were recruited. They were divided and randomized (intentionally and balanced) into two groups, 12 participants per group. The randomization method was performed using an opaque bag with black and white paper. The first subject took a piece of paper from the bag: if it was black, the patient would be assigned to the control group and placed on a waiting list to receive appropriate intervention at a later date. From there, the next subject was assigned to the experimental group and so on, alternatively.

As described in detail previously [5], subjects were included who experienced the presence of pain in the elbow region (≥3 months) and/or within 2 cm of the lateral humeral epicondyle on resisted extension of the wrist and could not practice sport due to the pain. Participants who had radicular pain, any previous surgery or acute trauma in the upper extremity, bilateral symptoms, electrophysiological findings referable to another peripheral nerve, were currently taking medication or receiving physiotherapeutic treatment (massage, therapeutic exercise and electrotherapy) (before and during the study), and patients exercising during treatment were excluded. 

### 2.3. Ethical Considerations

This study was approved by the local ethics committee, respecting all the principles of the Declaration of Helsinki. To participate in this study, patients needed to sign informed written consent forms. This trial was registered in the clinical trials database (clinicaltrials.gov) with registry number NCT03433716. 

### 2.4. Clinical Assessment

Demographic data were obtained including age, weight, height, body mass index (BMI), sex, the use of the injured arm, symptom duration and dominant side. Severity of average pain at palpation in the lateral epicondyle was evaluated using a numerical rating scale (NRS) (0 point, no pain; 10 points, maximum pain). Pain and functional ability over the previous week were evaluated using the Patient-Rated Tennis Elbow Evaluation (PRTEE) [31]. This questionnaire combines 2 parts: a pain scale composed of 5 questions and a functional scale consisting of 10 questions. Answers must be given on a scale graduated from 0 to 10 (0 represents the absence of pain or difficulty while performing a task and 10 represents the worst imaginable pain or the complete inability to perform a task). The maximum score for the first part is 50 and for the second part 100. To calculate the total score, the disability score is divided by 2 and is added to the pain score to get a score out of 100. 

### 2.5. Ultrasonographic Evaluation

**Figure 1:**
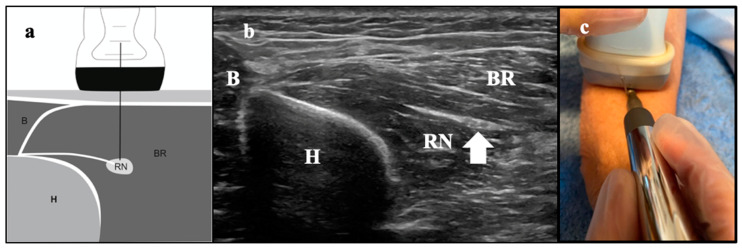
US-Guided PNM: (**a**) PES intervention in the radial nerve; (**b**) Ultrasound image of the intervention. B: brachialis muscle; BR: brachioradialis muscle, RN: radial nerve, H: humerus; and (**c**) ultrasound-guided invasive approach of the radial nerve.

All ultrasonographic examinations were scanned by an experienced therapist (over ten years in musculoskeletal US). A 7–12-MHz linear array transducer (Logiq e, GE, Medical Systems, New York, NY, USA) was used. The radial nerve cross-sectional area (CSA) was measured just before the bifurcation of the nerve, at 4 cm proximal to the tip of the lateral epicondyle of the humerus [5,32,33]. As described in detail previously [5], all the participants were seated while their arms were supported on a table, forearms pronated and elbows flexed 90°. CSA of the radial nerve was estimated by tracing a continuous line around the inner borders of the hyperechoic rim (Figure 1). A mean value of three consecutive measurements was recorded. The image analysis was performed by one researcher, blinded to the diagnosis, using ImageJ software (v.1.48 (2018), National Institutes of Health, Bethesda, MD, USA). The intra-rater reliability, measured with the intra-class coefficient correlation (ICC), was calculated in 10 subjects as satisfactory for clinical measurement. The ICC value was 0.81 (0.58–0.91), indicating high reliability.

### 2.6. Strength-Duration (S-D) Curves 

The S-D curve [34] is a traditional test of nerve excitability that is based on the relationship between the intensity of an electric monophasic rectangular pulse and its duration in causing a motor response when a motor nerve is electrically stimulated [35]. The result of this test is a hyperbolic function [35]. For the analysis, the subject lay supine with a pillow under their head. The studied forearm was bare and the skin was shaved and cleaned prior to placement of the electrodes. A blinded and experienced physiotherapist (10 years of experience in classical electro-diagnosis) performed the S-D curves with a constant current stimulator (Myomed 932; Enraf Nonius Co., Rotterdam, The Netherlands). This device is characterized by an intensity of current that can be increased with an accuracy of 0.1 mA from 1 to 15 mA, with an accuracy of 0.5 mA from 15 to 30 mA, and with an accuracy of 1 mA from 30 to 80 mA (maximal intensity). One 24 cm^2^ (6 × 4 cm) ground electrode (anode) was fixed with a Velcro strap on the first third proximal of the forearm, and another 24 cm^2^ (6 × 4 cm) active electrode (cathode) was placed on the second third proximal of the forearm. The first value of S-D curves obtained was the rheobase, with a monophasic rectangular pulse duration of 1000 ms and a between-pulse rest of 1 s. The current intensity was increased until the observation of a minimal visible twitch contraction of the epicondylea musculature (innervated by the radial nerve). Then, the S-D curve was obtained by successively decreasing the durations of the rectangular pulses and observing the current threshold for maintaining a minimal visible twitch contraction of the muscle. When the S-D curve obtained using rectangular pulses was finished, the S-D curve made using triangular pulses was obtained in the same manner. Between the 2 tests, the active electrode was moistened again and replaced in the same position that it was in during the previous curve. The chronaxie and accommodation index (AI) were analyzed [35]. Chronaxie is the impulse time needed to obtain a minimal muscle contraction with a monophasic rectangular impulse and intensity twice the rheobase, measured in milliseconds, being the rheobase the intensity threshold needed to obtain a minimal muscle contraction with a monophasic rectangular impulse and a width of 1000 milliseconds (ms), measured in mA. The normal value of chronaxie is between 0.1 and 1 [35]. The accommodation index is the quotient between the intensity thresholds at 1000 ms triangular and 1000 ms rectangular impulses (adimensional). The normal value of the accommodation index is greater than 3. A minor excitability of the nerve is characterized by values lower than 3 [36].

### 2.7. Therapeutic Intervention

The subjects in the US-guided PNM group received the PES (percutaneous electrical stimulation) intervention. As described earlier, subjects randomised to the control group were placed on a waiting list for treatment, therefore at the time of the study, these subjects did not receive any interventions. PNM protocol in the different studies is varied [13,23,24,25,26,27,28,29]. Most studies analyzed the effect of one single intervention on pain, range of motion or muscle strength and used low frequency (2–10 Hz) and high pulse width (240–250 µs). According to a previous case report [14] and the clinical experience of the authors, subjects were asked to attend once a week for 3 weeks. Specifically, this procedure was based on the employment of a square wave biphasic electrical current, with a 10 Hz frequency, a 250-µs pulse width, and sufficient intensity to cause a visible muscle contraction for a total of 1.5 min. Certified equipment (Physio Invasiva^®^, PRIM Fisioterapia y Rehabilitación, Spain) was applied to the subjects lying supine, with forearms pronated and elbows moderately flexed. The radial nerve was located at 4 cm proximal to the tip of the lateral epicondyle of the humerus using an ultrasound machine (cross-section) (Logiq e, GE Healthcare, New York, NY, USA) with a high-frequency linear US transducer 12 L, and subsequently, a stainless-steel acupuncture needle (0.30 mm × 30 mm) (Physio Invasiva^®^, PRIM Fisioterapia y Rehabilitación, Spain) was inserted in a short axis approach, perpendicular to the skin, up to the perineurium of the radial nerve (Figure 1). Prior to inserting the needle, isopropyl alcohol was used to clean the underlying skin. The PES intervention was administered by an experienced physiotherapist (over 10 years) in US-guided PNM therapy. 

All variables were measured at the baseline and at one month in both groups. This means that for the experimental group, the post-treatment measure was one week after the last intervention.

All participants had to continue with the same lifestyle that they had before starting the study.

As described in detail previously [37], all patients were asked to report any adverse events that they experienced during the research. 

### 2.8. Statistical Analysis

All variables were expressed as mean and standard deviation. All data were checked for normality using the Shapiro–Wilk test, after a descriptive analysis. The homogeneity of variances was observed using Levene’s test. Linearity was evaluated with bivariate scatter plots of observed residual values against the expected values. To check the baseline at pre and post intervention for each variable between groups a t test for independent samples was employed. Comparisons between both groups were conducted for clinical data using an analysis of variance (ANOVA) with one between-group factor (control group versus PNM group). Effect sizes (ES) were also calculated using partial Eta^2^ d coefficient. Data were analyzed with the Statistical Package for the Social Sciences (SPSS) v.21 (SPSS Inc., Chicago, IL, USA) and statistical significance was set at *p* < 0.05. Moreover, the intraclass correlation coefficient (ICC) was calculated to evaluate the intra-rater reliability of all the ultrasound measurements.

## 3. Results

There were no significant baseline differences between both groups in any of the clinical variables (Table 1). Regarding pain level, PRTEE scores and CSA values are summarized in Table 2. There were no differences between groups in the baseline measurements (all *p* > 0.05). However, at the end of the study, significant differences were reported as only the PNM group significantly improved their values compared to baseline (all comparison *p* = 0.001).

S-D curves for the radial nerve revealed different results (Table 2). Chronaxie values were all normal and similar between the control and PNM groups (pretest: 0.74 ± 0.2 vs. 0.70 ± 0.2; posttest: 0.72 ± 0.1 vs. 0.60 ± 0.2, respectively; *p* > 0.05 for all comparisons). The ICC was 0.80 (0.56–0.91) for chronaxie values indicating high reliability. Regarding the AI, both groups showed nerve hipoexcitability at the beginning of the study (AI < 3). After the study, only the PNM group showed significant improvement in the pattern of the electrophysiologic excitability of the nerve, returning to the normal function in all radial nerves (AI > 3; *p* < 0.001). The ICC was 0.70 (0.33–0.86) for accommodation indices indicating moderate reliability.

## 4. Discussion

The purpose of this study was to calculate the effects of an US-guided PNM in patients with chronic unilateral LE. The data from this preliminary study demonstrate that PNM may be safe and generally well tolerated for many patients with moderate to severe symptoms. In fact, there were no adverse events and symptoms, such as post-puncture pain. PNM-treated patients demonstrated decreased pain scores, increased functional ability, decreased radial nerve CSA and improved electrophysiologic excitability of the radial nerve. According to the previous case report [14], these results confirmed our initial hypothesis that percutaneous neural therapy may provide a substantial effect on unilateral LE. To date, this is the first clinical trial study to demonstrate the effect of three sessions of US-guided PNM technique on nerve morphology and excitability in LE patients.

Current evidence suggests that the lesional mechanism of LE is complex and the result of an interplay of three different components: local tendon pathology, changes in the pain system (sensitization), and motor system impairment (dysfunction) [38]. Whereas the degree of local tendon changes and motor system impairment may be assessed by ultrasonography and physical examination, it is more complicated to assess changes in the pain system. This is why the diagnosis of LE is based upon clinical examination and indeed, authors found in this study that LE caused pain (focus symptom) at both resting and functional levels, measured with a PRTEE questionnaire (Table 2). However, there are already authors who highlight the ultrasound as a good tool for diagnosis and evaluation of treatment in LE patients [5,39,40] as well as the S-D curve as a procedure for analysis the nerve excitability [34]. 

Ultrasound findings in elbow tendon pathology have been well reported with both structural [41] and blood flow changes described [42]. However, it´s not clear which findings have the greatest impact on clinical outcomes such as are symptoms and performance [43,44]. Current studies have proposed an association between neovascularity and pain and suggested that an adequate clinical outcome is related with improvement in neovascularity rather than structural changes [45,46]. Clarke et al. [41] suggested that the degree of tendon injury is the main factor in the prognosis of LE. Bisset et al. [47] stated that LE is characterized by locally increased facilitation of pain, as measured by temporal summation, but this is not associated with severity of pain or disability. Gurcay et al. [5] supported the idea that some of the chronic complaints of these patients might actually be due to radial nerve entrapment. This is the reason why the authors did not want to take the tendon condition into account. They wanted to analyze the nerve’s involvement in elbow pain. In this line, researchers hypothesized that LE pathology may be characterized by a structural and excitability alteration of the radial nerve, compatible with the presence of pain and or not with the presence of tendon alterations. So, the authors found the patients had a high CSA value of the radial nerves and they presented a lower excitability too, measured using the AI. Electrodiagnostic studies have been used for evaluating the radial nerve involvement in radial tunnel syndrome: some authors have found normal or insignificant changes in the affected side, while others have shown increased radial nerve distal motor latency during forearm supination, or neuropathic changes revealed by electromyography in some patients [48,49]. Generally, the curve of a degenerative process is characterized by an increased stimulation threshold and a lack of response to shorter duration pulses and the result is a lower AI [34,50]. At the beginning of our study, the authors found abnormal electrophysiological findings in all patients and there were no differences between groups. After the intervention, all patients treated that had a pattern of lower excitability of the radial nerve, attained the normal function in all nerves. The S-D curve is an electrofunction modality that may be used as an adjunct to ultrasound for the evaluation of peripheral nerve problems. 

According to these results, authors suggest that the treatment of the LE pathology should be based on the tendon and the radial nerve and that neural therapy, based on US-guided PNM, may be an important treatment in LE patients. US guided-PNM is less invasive than lead implantation and looks far less dangerous than deep brain stimulation [51] or motor cortex stimulation [52]. The use of ultrasound guidance has been developed to decrease the risk of nerve injury and to improve tolerance to neurostimulation. The authors believe that US-guided PNM of the radial nerve combines the efficacy of upper limb PNS with lower invasiveness and better tolerance.

### 4.1. Clinical Implications

The main clinical implication of the present study is that the three-session US-guided PNM treatment on the painful elbow produces effects, not only on the pain but also on the nerve morphology and excitability in LE patients. The relevance of this finding for clinical practice is that this invasive neurostimulation technique may be proposed to treat chronic pain of the lateral elbow resulting from peripheral radial nerve damage, and to apply it therapists must be fully prepared and trained. The aim of the neuromodulation intervention was to decrease the sensitization of the radial nerve. Different potential mechanisms may explain these effects, due to the stimulation of large-diameter, myelinated, afferent peripheral nerve fibers which may not allow communication of nociceptive signals to the central nervous system from small-diameter pain fibers at the level of the spinal cord (gate control theory) [53]. According to this theory, although the results of this study do not provide information on the mechanisms underlying the observed changes, they suggest that the US-guided PNM technique may significantly improve the painful elbow and may cause changes in radial nerve morphology and excitability in patients with LE.

### 4.2. Study Limitations

The current study presented some limitations. Firstly, PNM therapy was applied independently; nevertheless, clinical physical therapists usually treat their LE patients with different techniques. Future studies should examine the effectiveness of several methods including PNM therapy in combination with other accepted techniques. Secondly, authors did not consider the ultrasound characteristics of the common extensor tendon as a study variable. It would be of great interest to analyze the involvement of the nerve based on the pathological characteristics of the tendon. Third, pain level was calculated with NRS. It would be of interest to use other methods to analyze this variable, such as an algometer. Fourth, the sample size was small and there was no evaluation of a follow-up period after the treatment. However, these findings may provide preliminary data that could allow a future methodological recommendation to use this procedure to treat patients with chronic LE.

## 5. Conclusions

In conclusion, in the light of these results, the authors propose that the US-PNM technique may be an interesting therapeutic tool for the treatment of chronic LE due to the improvement in the level of pain, functionality, and nerve morphology and excitability in this population. Three-session US-guided PNM may be a suitable tool for treatment of this pathology.

## Figures and Tables

**Table 1:** Sociodemographic data and clinical features.

Characteristic	Total Sample (N = 24)	Control Group (N = 12)	PNM Group (N = 12)	*p* Value
Age (years)	49.4 ± 7.6	49.4 ± 5.5	49.5 ± 9.5	0.979
Weight (Kg)	68.6 ± 12.13	66.83 ± 11.16	70.41 ± 13.2	0.482
Height (cm)	170.5 ± 9.6	170.00 ± 9.85	171.6 ± 8.7	0.808
BMI (kg/m^2^)	23.44 ± 2.5	22.98 ± 2.23	23.8 ± 2.80	0.392
Gender (F/M)	12 F/12 M	6 F/6 M	6 F/6 M	n/a
Use of the affected arm (R/L)	24/0	12/0	12/0	n/a
Hand dominance (R/L)	24/0	12/0	12/0	n/a
Duration of Symptoms (months)	13.6 ± 9.10	12.4 ± 8.06	14.9 ± 10.2	0.513

Data given as mean ± SD. PRTEE: Patient-Rated Tennis Elbow Evaluation scores; CSA: cross-section area. * Between-groups statistical significance (one factor-ANOVA). *p* values (*p* < 0.05).

**Table 2:** Clinical features, Patient-Rated Tennis Elbow Evaluation (PRTEE) scores, cross-section area (CSA) scores and chronaxie and accommodation index (AI) intrasubject effects.

			Intrasubject Effects	
Measure	PNM Group (n = 12)	Control Group (n = 12)	Time Value F (Df); P (Partial Eta^2^)	Treatment X Time F (Df); P (Partial Eta^2^)
NRS (point)			F (1, 22) = 102.47; *p* = 0.001 (0.823)	F (1, 22) = 105.36; *p* =.001 (0.827)
Baseline	7.7 ± 0.9	7.6 ± 1.15		
Post-Treatment ^†^	1.7 ± 1.8	7.6 ± 1.0		
PRTEE (total score)			F (1, 22) = 40.28; *p* = 0.001 (0.647)	F (1, 22) = 54.73; *p* =.001 (0.713)
Baseline	83.3 ± 22.4	75.6 ± 22.6		
Post-Treatment ^†^	38.6 ± 22.2	79.0 ± 25.2		
PRTEE (pain score)			F (1, 22) = 24.06; *p* = 0.001 (0.522)	F (1, 22) = 17.609; *p* = 0.001 (0.445)
Baseline	28.5 ± 7.0	29.6 ± 6.7		
Post-Treatment ^†^	15.75 ± 7.8	28.6 ± 7.2		
PRTEE (functional subscale-specific activities)			F (1, 22) = 18.62; *p* = 0.001 (0.458)	F (1, 222) = 29.28 *p* = 0.001 (0.571)
Baseline	30.9 ± 13.8	26.9 ± 13.0		
Post-Treatment ^†^	13.1 ± 10.7	28.9 ± 14.4		
PRTEE (functional subscale-usual activities)			F (1, 22) = 38.30; *p* = 0.001 (0.635)	F (1, 22) = 58.78 *p* = 0.001 (0.728)
Baseline	23.0 ± 6.0	21.4 ± 7.8		
Post-Treatment ^†^	10.5 ± 7.3	22.7 ± 5.9		
CSA (mm^2^)			F (1, 22) = 42.06; *p* = 0.001 (0.657)	F (1, 22) = 39.60; *p* = 0.001 (0.016)
Baseline	0.74 ± 0.13	0.69 ± 0.1		
Post-Treatment ^†^	0.57 ± 0.1	0.69 ± 0.1		
Chronaxie			F (1, 22) = 1.03; *p* = 0.319 (0.045)	F (1, 22) = 0.55; *p* = 0.463 (0.025)
Baseline	0.70 ± 0.2	0.74 ± 0.2		
Post-Treatment ^†^	0.60 ± 0.2	0.72 ± 0.1		
AI			F (1, 22) = 15.88; *p* = 0.001 (0.419)	F (1, 22) = 22.59 *p* = 0.001 (0.507)
Baseline	2.35 ± 0.5	2.45 ± 0.4		
Post-Treatment ^†^	3.11 ± 0.5	2.39 ± 0.5		

Values are mean ± SD unless otherwise indicated. ^†^ Significant differences (*p* < 0.05) between PNM group and control group. Abbreviature: AI, accommodation index; CSA, cross-sectional area; NRS, numerical rating scale; ROM, range of motion.

## Data Availability

The data presented in this study are available on request from the corresponding author. The data are not publicly available due to ethical condition.
